# The Use of Botulinum Toxin as Primary or Adjunctive Treatment for Post Acne and Traumatic Scarring

**DOI:** 10.4103/0974-2077.69019

**Published:** 2010

**Authors:** Greg J Goodman

**Affiliations:** *Monash University, Clayton Victoria, Australia*

**Keywords:** Post acne, Botulinum toxin, scar, ageing skin

## Abstract

**Background:**

Botulinum toxin has been utilised successfully in many facial and extra facial regions to limit superfluous movement. Scars, whether traumatic or disease-related, are treated with many modalities.

**Objective:**

To assess the available literature concerning the prophylactic use of botulinum toxin for the improvement in the cosmetic outcome of scars induced by surgery and to examine its role in the treatment of established scars alone, as also combined with other modalities.

**Material and Methods:**

The results of the prophylactic use of botulinum toxin to limit the resultant scarring from surgery are examined by a literature review. The primary and adjunctive use of botulinum toxin in the treatment of post acne and post surgical and traumatic scars is explored by case examples.

**Results:**

Literature review and personal experience shows good Improvement in the appearance of scars with the use of botulinum toxin alone or with other adjuvant modalities in the treatment of scars.

**Conclusion:**

Botulinum toxin would appear to be useful both in the prophylaxis and treatment of certain types of scars.

## INTRODUCTION

Botulinum toxin has been used for over 20 years in the correction of many diverse aesthetic and non-aesthetic problems. These include strabismus, nystagmus, achalasia, anal fissue, dystonias including cerebral palsy, torticollis, dysphonia, blepharospasm, hemifacial spasm[1,2] and hyperhidrosis.[3] Botulinum toxin now also has a long and stable history in the treatment of facial hyperkinetic lines.[4–8]

The treatment of facial scarring is rarely a monodimensional process. Scarring from acne or trauma is treated by various means and is often combined with other treatments. Subcision, punch techniques and excision are often used prior to or concurrently with resurfacing or fractional resurfacing treatments. The role of botulinum toxin in the treatment of scarring should be seen in this light, as an adjunctive treatment. Such a combination approach with botulinum toxin is not new, as a similar approach has been used to improve the outcomes from other techniques, including fillers,[9] intense pulsed light treatments[10] and resurfacing.[11] Botulinum toxin is also often utilised as part of an overall ‘rejuvenation’ programme.

Where and when can one consider botulinum toxin in the treatment of scarring? Botulinum toxin can play a role in both prophylaxis and treatment of scars.

## BOTULINUM TOXIN IN PROPHYLACTIC USE FOR SCARS

A scar is unacceptable if it is long, hypertrophic, or sharply defined and atrophic. It is also unacceptable if it runs against the natural relaxed skin tension lines or crosses cosmetic boundaries. On occasions, a scar is present and requires revision or sometimes a lesion requires excision and there is a need to plan the best outcome. A scar may not naturally conform to the best direction of the relaxed skin tension lines of the face or body. Although excision techniques such as Z or W plasty are useful in reorientation of scar direction, they are not always completely effective alternatives and may create extra scarring on the patient. In such situations, botulinum toxin may be used to eliminate the tension produced by muscular forces surrounding the scar.

It would probably be the best practice in these circumstances to inject superficially at the time of the procedure or at suture removal and in a low dose (probably 5 units of Botox^®^ would suffice in most circumstances depending on the size of the scar), so as not to compromise deeper muscular activity, especially when this is not in a midline or typically in an area injected with botulinum toxin, such as, the cheek. There would probably be less concern on most areas of the body.

In addition to botulinum toxin’s well-known effect on reducing muscular activity there also appears to be an inhibitory effect of botulinum toxin itself on fibroblasts,[12] thereby, offering another potential mechanism for producing a more satisfactory outcome for problematic scar revision and lesion removal.

Botulinum toxin has been studied in the prevention of scarring in animal models, following traumatic wounding. It has been shown in animal testing that, botulinum toxin prevented increase in collagen content during urethral wound healing.[13] Intra-articular botulinum toxin has been demonstrated to be useful in preventing arthrofibrosis in rabbits after transection of the anterior cruciate ligament.[14]

In a series of articles, researchers at the Mayo Clinic initially showed favorable results in a randomised, double-blind, placebo-controlled primate study, with symmetric pairs of standardised excisions performed on either side of the forehead in six primates. The half foreheads that were injected with botulinum toxin had a significantly better appearance than their placebo counterparts.[15] Further articles have tended to show improved outcomes of scars treated with botulinum toxin in humans.[16–19]

## TREATMENT FOR THE APPEARANCE OF ESTABLISHED SCARS

Occasionally, scarring will appear in areas that are amplified by movement. This may be less obvious when one is young. However, as one ages, tissues become less supple and the skin diminishes in thickness allowing the underlying muscular activity to become more apparent as wrinkles and lines. If one is scarred, particularly if this scarring is atrophic or mildly hypertrophic, normal muscle movement may have an exaggerated effect on normal ageing skin. This seems to be particularly true of rolling scarring[20] or grade 3 atrophic scarring[21] (scarring that is visible at a conversational distance, but which disappears on manually stretching the skin). The movement related to facial expression continues unabated throughout life, but as we age the resistance of dermal tissues that have suffered the twin destructive elements of time and scarring, struggle to resist such movements, resulting in more a prominent visibility of scars. In such areas, botulinum toxin may be employed to the cosmetic advantage of the patient, although in some areas of the face, such as the cheeks, the role of botulinum is limited by potential side effects.

**Figure 1:**
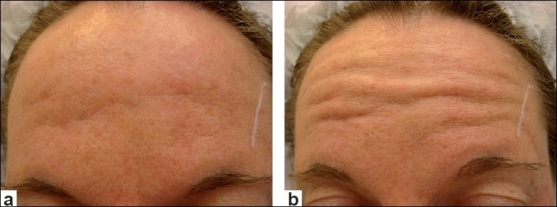
(a) Forehead scars at rest; (b) Forehead scars contracted

**Figure 2:**
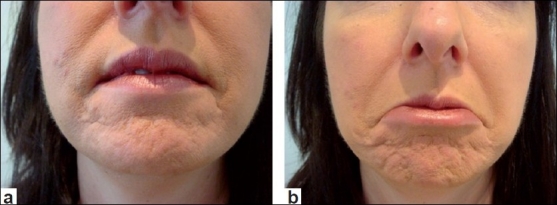
(a) Lower face at rest; (b) Mentalis and depressor anguli oris (DAOs) contracted

**Figure 3:**
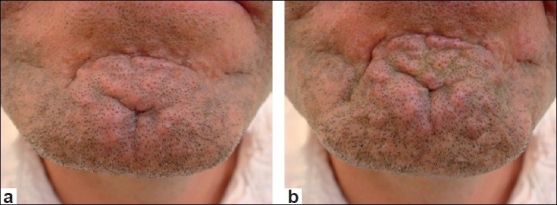
(a) Chin hyperplastic scarring at rest; (b) Chin hyperplastic scarring contracted

In the upper face, the forehead [Figures 1a and b] and glabellar areas may be affected locally by acne, traumatic or surgical scarring; and would be suitable targets for botulinum toxin to improve their day-to-day appearance. In the lower face, the marionette lines [Figure 2a and b] and chin [Figure 3a and b] are the two most commonly affected areas that lend themselves to botulinum toxin treatment. The chin and surrounding areas seem to be areas that cope badly with acne in particular and also appear to age badly once affected. Sometimes mildly hypertrophic scars in these areas may be exaggerated by movement [Figure 3a and b]. In the lower face, scars may occasionally be influenced by other movements such as pursing of the lips and although more difficult to treat, these could be the targets for the judicious use of botulinum toxin.

**Figure 4:**
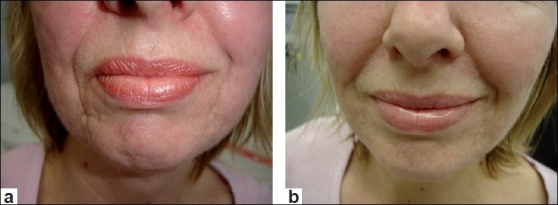
(a) At rest, prior to botulinum toxin for DAOs, depressor labii inferioris (DLI), mentalis and fillers; (b) At rest, post botulinum toxin for DAOs, DLI, mentalis and fillers

**Figure 5:**
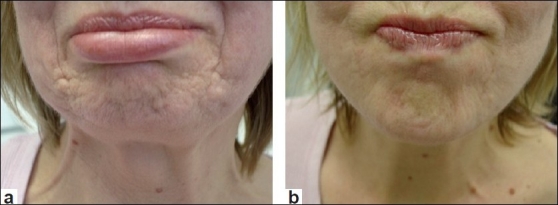
(a) Mouth frown prior to botulinum toxin for DAOs, DLI and Mentalis and dermal fillers; (b) Mouth frown post botulinum toxin for DAOs, DLI and Mentalis and dermal fillers

Botulinum toxin may be combined with fillers at the same session, or as is more common, fillers are employed two weeks later, once the effect of botulinum is established. Over a period of time the recurrent action of the muscles on the skin is weakened by atrophic scarring and may force the skin to fold unnaturally, accentuating the scars. As the atrophic skin poorly resists the muscular forces, the natural folds become unnatural and gives a prematurely aged appearance. If this continues static lines will be forced into the skin, becoming deeper over time. Releasing the action of these muscles may immediately help the appearance of the skin [Figures 4a, b and 5a, b]. Dermal fillers may further augment this process by targeting these etched lines if the botulinum toxin is not sufficient to soften the appearance of the scars completely. Occasionally muscles may be targeted to improve abnormalities in facial expression not related to facial scarring. The use of dermal fillers and botulinum toxin appears to be synergistic in many cases.[9]

In all the above cases used as examples, the botulinum toxin used was Botox^®^ (Allergan Inc, Irvine CA).

## DISCUSSION

Botulinum toxin may act to improve the outcome of scars in a number of ways. Botulinum may have an inhibitory effect on scar formation; it may assist in a prophylactic manner in excisions where the resultant scar is likely to contrast against relaxed skin tension lines or where a hypertrophic scar is to be re-excised. For existing traumatic or post acne scarring, botulinum has a role in treating scars that happen to lie in an area where they are exaggerated by movement and there will be no untoward cosmetic effect on the patient. Scarring in the upper face (forehead, periorbital, glabella) and the lower face (chin and surrounding areas) tends to be well-targeted by botulinum either alone or more usually in concert with other techniques such as fillers, resurfacing and surgery.
